# Impact of novel herbicide based on synthetic auxins and ALS inhibitor on weed control

**DOI:** 10.1515/biol-2022-0868

**Published:** 2024-04-26

**Authors:** Monika Grzanka, Andrzej Joniec, Janusz Rogulski, Łukasz Sobiech, Robert Idziak, Barbara Loryś

**Affiliations:** Agronomy Department, Poznań University of Life Sciences, Wojska Polskiego 28, 60-637 Poznan, Poland; Ciech Sarzyna S.A., ul. Chemików 1, 37-310 Nowa Sarzyna, Poland

**Keywords:** MCPA, tribenuron-methyl, winter cereals, weed control, spring application

## Abstract

Delayed sowing of winter cereals or unfavorable weather conditions in autumn may make it impossible to carry out herbicide treatment in autumn. In such cases, weed control should be started in the spring. During this time, the plantation should be protected as effectively as possible because the weeds are at an advanced stage of growth. Therefore, they are less sensitive to applied herbicides. In the treatment, it is worth using a mixture of different mechanisms of action. Studies were conducted to evaluate the effectiveness of a band of tribenuron-methyl, and MCPA applied as soluble granules in spring control of dicotyledonous in winter cereals. The biological efficacy of herbicides was estimated in the 25 field experiments on winter cereals in Poland. Postemergence, a spring application of tribenuron-methyl + MCPA, effectively controls the majority of weed species present in spring: *Anthemis arvensis*, *Brassica napus*, *Capsella bursa-pastoris*, *Centaurea cyanus*, *Lamium purpureum*, *Matricaria chamomilla*, *Tripleurospermum inodorum*, *Stellaria media* and *Thlaspi arvense*. Satisfactory control was confirmed for *Veronica persica*, *Viola arvensis,* and *Galium aparine*. Tribenuron-methyl with MCPA is recommended for application to winter cereals in spring. To prevent the development of resistance in weeds, it is advantageous to combine two active substances.

## 1 Introduction

Crop yields depend on many elements, including the genetic potential of productivity, habitat conditions, and agrotechnical factors [1]. However, yields can be significantly reduced by the occurrence of weeds, pests, and diseases [2]. When assessing the level of weed infestation in arable fields, it is also worth determining species diversity [3]. Individual species of plants side effects do not act independently of each other and form certain communities [4]. The species composition of weeds in an area is affected by many factors, including the type of climate, soil structure, pH, and topography [5]. Chemical plant protection is of great importance in maintaining the proper yield of crops. It is one of the elements of modern agriculture and a prerequisite for maintaining food security [6]. Herbicides show high specificity to the place they act. This became the key to their classification [7].

One of the main groups of crop protection products is herbicides with actions similar to natural auxins. Auxins are the best-studied group of phytohormones, playing a leading role in regulating a number of plant growth and development processes. Natural auxins such as indole-3-acetic acid are involved in cell division, vascular tissue and floral meristem differentiation, leaf initiation, production of root buds, phyllotaxy, and tropism reactions, among others. For the proper functioning of the plant organism, hormones should remain in balance – any changes in the content of one phytohormone affect the functioning of the next [8]. The use of synthetic auxins causes plant reactions that are characteristic of a concentration of natural phytohormones that are too high in their organisms [9]. Herbicides based on synthetic auxins are used to control dicotyledonous weeds in cereal crops, golf courses, and lawns, as well as in horticulture, forestry, aquatic environments, and non-agricultural areas. Symptoms of the action of these herbicides are tissue thickening, curling of stems, inhibition of growth, chlorosis, and necrosis of plants [10,11]. Examples of substances belonging to this group are MCPA and dicamba [12]. MCPA (CAS number 94-74-6) is classified as a phenoxyalkane herbicide. It is a derivative of acetic acid, linked at position 2 to a 4-chloro-2-methylphenoxy group [13,14,15]. In its chemically pure state, it is a colorless crystalline substance with no odor. In contrast, the technical product is white in color and has a characteristic odor associated with the admixture of phenols. Due to its high hydrophobicity, that is, its low affinity for water molecules, MCPA is practically insoluble in water. Its solubility in water is 825 mg L^−1^ at 20°C [16,17].

Tribenuron-methyl (CAS number 101200-48-0), with the common name 2[4-methoxy-6-methyl-1,3,5-triazin-2-yl(methyl)carbamoylsulfamoyl]methyl benzoate, belongs to the group of triazinosulfonylurea herbicides. Herbicides in this group are characterized by a broad spectrum of action, so they are widely used in the control of dicotyledonous broadleaf weeds in cereal crops [18,19]. In terms of chemical structure, tribenuron-methyl is a methyl ester of tribenuron [20]. This compound is well soluble in water (2,040 mg L^−1^ at pH 7, at 20°C), while it is poorly soluble in organic solvents. The technical product is in the form of a powder of light beige color with a slightly pungent odor. According to the HRAC classification, tribenuron-methyl is classified as a class 2 (HRAC)/B (Legacy HRAC) inhibitor of acetolactate synthase (ALS, EC 4.1.3.18). ALS, also referred to as acetohydroxyacid synthase, is the first enzyme in the biosynthesis pathway of the branched-chain amino acids valine, leucine, and isoleucine. ALS catalyzes the condensation reaction of two pyruvate molecules to 2-acetolactate, which is an intermediate product in the biosynthesis of valine and leucine [21,22,23]. Three to four weeks after the application of these substances, chlorosis, tissue necrosis, and plant death occur [24]. Literature data indicate that the widespread use of ALS-inhibiting herbicides leads to the emergence of resistant weed biotypes [25,26]. The application of herbicides with different modes of action is one of the basic methods of preventing weed resistance [27].

Pesticide formulations do not consist of active ingredients alone. In addition to the active substances, the products available in the market contain a combination of a number of auxiliary ingredients and fillers to give a specific formulation. Each type of formulation has its advantages and disadvantages. For example, wettable powders tend to dust during handling, which poses a serious risk to the user. In order to improve the quality and safety of pesticides, more improved types of formulations continue to be introduced into the chemical industry. Modern pesticide formulations mainly include suspension concentrates, oil-in-water emulsions, microcapsules, and soluble granules (SG) [28]. SG have gained an advantage over the other forms, mainly because they can be formulated for the most known active ingredients [29]. In this form, the active ingredient is trapped in granules that are completely soluble in water. Granules can be produced by a number of methods, with a distinction between dry and wet granulation [30]. Extrusion granulation, fluidized-bed granulation, or high-speed granulation technologies are most commonly used to produce SG [31,32,33].

Weed control should be carried out when it poses the greatest threat to the crop to protect the high yield potential of modern crop varieties [34]. In practice, regardless of the crop species, the treatment is carried out in the first weeks after sowing, as this is when the plants are most exposed to strong competition from weeds [35]. However, climatic changes very often result in abnormal weather patterns in the autumn following the sowing of winter cereals that favor weed growth and make it difficult, if not impossible, to control them at this time. The solution is to control weeds in the spring, which in turn is made more difficult because of the strongly developed weeds [36]. In the working hypothesis, it was assumed that the spring application of a mixture of herbicides with different mechanisms of action with the addition of an adjuvant would make it possible to effectively control dicotyledonous weeds from the winter wheat.

In this study, we performed a series of 25 field experiments in Poland with the aim of examining the potential of herbicide combination as SG containing 15 g kg^−1^ tribenuron-methyl and 550 g kg^−1^ MCPA. This herbicidal product is intended to be used in spring for the control of dicotyledonous weeds that have already emerged in autumn in winter cereals.

## 2 Methods

The production of MCPA + tribenuron-methyl 565 SG uses dry granulation technology by extrusion or high-speed mixing. The first stage of the production process is the preparation of the mixture of ingredients and their homogenization in a pre-mixer. In order to achieve the desired grain size (100% particles less than 10 µm), the raw materials are ground on a jet mill. The milled mixture is then mixed with a hopper, which can be water or water with surfactants. The use of a twin-screw extruder allows water to be dosed concurrently with the powder and the ingredients to be mixed very thoroughly, which is an advantage for SG. The resulting product mass is then extruded through the openings of the head located at the outlet of the machine. In the case of granulation with a high-speed mixer, granules are formed by agglomerating bulk materials while simultaneously dosing powder and water. The granules produced by one of the two techniques are successively directed to a fluidized bed dryer until the required moisture content is achieved. The final step is to sieve the resulting material to separate the sub-grains and super-grains.

**Figure 1:**
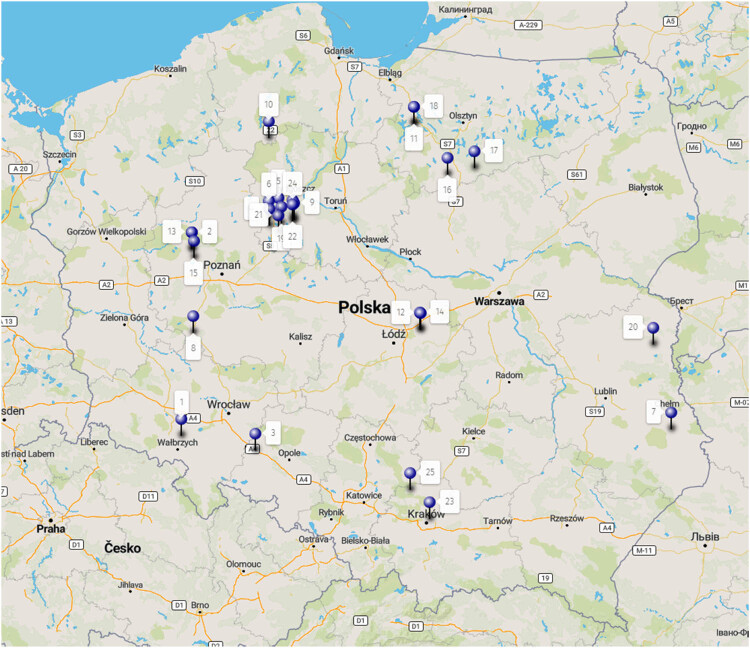
Locations of the experimental fields in Poland.

Field experiments were conducted in Poland in 2016 and 2017. A total of 25 efficacy trials were carried out on a range of several cultivars for winter wheat, winter barley, winter rye, and winter triticale. Trial locations of the experimental fields are presented in Figure 1 and Table 1.

**Table 1:** Experimental site description and application details

Trial no.	Crop species; variety	Application date; crop stage (BBCH)	Soil texture (pH)	Water volume at application (L ha^−1^)
1	Winter wheat; Bamberka	24/04/17; 23	Sandy loam, 5.8	250
2	Winter wheat; Muszelka	24/04/17; 31	Sandy loam, 6.5	300
3	Winter barley; Joy	21/04/17; 31	Silty loam, 6.5	300
4	Winter rye; Dankowskie Zlote	20/04/17; 15	Loamy sand, 6.0	300
5	Winter rye; Dankowskie Diament	20/04/17; 25	Loamy fine sand, 6.7	300
6	Winter barley; Souleyka	11/04/17; 14	Clayey sand, 6.7	250
7	Winter wheat; Ozon	05/04/17; 13	Sandy clay loam, 6.1	200
8	Winter rye; Brasetto	21/04/17; 24	Sandy loam, 5.8	300
9	Winter wheat; Fidelius	10/04/17; 24	Loamy fine sand, 6.5	200
10	Winter triticale; Borwo	03/04/17; 14	Sandy loam, 7.1	200
11	Winter triticale; Twingo	03/04/17; 21	Sandy loam, 6.3	200
12	Winter wheat; Tonacja	11/05/17; 39	Sandy loam, 6.2	300
13	Winter barley; Bartosz	17/04/17; 28	Sandy loam, 5.4	250
14	Winter rye; Palazzo	05/05/17; 23	Sandy loam, 5.8	300
15	Winter rye; Horyzo	19/04/17; 30	Sandy loam, 6.3	250
16	Winter triticale; Gringo	20/04/17; 23	Sandy loam, 5.0	300
17	Winter triticale; Pisarro	03/05/17; 29	Loamy sand, 6.4	250
18	Winter triticale; Arktis	20/05/17; 37	Sand, 5.3	300
19	Winter barley Titus	20/04/17; 27	Sandy loam, 6.8	300
20	Winter barley; Sandra	11/04/17; 24	Sandy loam, 6.4	200
21	Winter wheat; Sailor	25/04/17; 25	Sandy clay, 6.8	300
22	Winter wheat; Jantarka	29/04/16; 31	Loamy fine sand, 7.3	250
23	Winter wheat; Bogatka	10/05/16; 32	Silty clay, 5.8	250
24	Winter rye; Daran	25/04/16; 32	Loamy fine sand, 3.1	250
25	Winter rye; Dankowskie Zlote	30/04/16; 32	Silty clay, 6.2	250

Field experiments were implemented in fields sowed with commercial cereal cultivars, where crops are produced commercially and with a known history of infection of the weeds. In all experiments, the infestation was natural. Sites were selected to represent the range of agricultural and environmental conditions (including climatic conditions) likely to be encountered in practice in the area of potential use. Weed species growth stages during herbicide application and the weed composition of the experimental fields are listed in Table 2.

**Table 2:** Weed species growth stages during herbicide application

Trial no.	Weed species											
	ANTAR	BRSNN	CAPBP	MATIN	VIOAR	CENCY	LAMPU	STEME	THLAR	VERPE	GALAP	MATCH
	BBCH											
1	12	12	11	12	11	—	—	—	—	—	—	—
2	51	—	47	—	—	40	51	55	42	—	—	—
3	14	14	—	—	16	—	—	—	12	—	—	—
4	14	14	—	—	13	17	—	—	—	—	—	—
5	13	—	—	—	13	13	—	12	—	13	—	—
6	—	15	—	—	14	—	15	16	—	—	15	—
7	—	13	14	14	—	13	—	12	—	13	—	—
8	—	16	—	14	16	14	—	—	—	16	—	—
9	—	—	14	—	—	—	—	—	—	—	—	—
10	—	—	16	—	—	14	—	—	—	—	—	16
11	—	—	19	—	—	19	—	—	—	—	—	—
12	—	—	—	—	18	19	—	15	—	—	16	15
13	—	—	—	35	—	35	51		47	47	––	—
14	—	—	—	—	17	18	—	14	—	—	15	14
15	—	—	—	—	34	32	—	—	—	26	27	—
16	—		—	—	12	19	—	—	—	—	—	13
17	—	—	—	15	—	16	—	—	31	32	—	—
18	—	—	—	—	61	55	—	—	—	—	—	30
19	—	—	—	—	—	—	—	12	16	—	14	—
20	—	—	—	—	—	—	15	—	16	—	14	—
21	—	—	—	—	13	—	15	12	—	13	—	—
22	—	—	—	—	51	—	—	—	—	—	—	—
23	—	—	—	—	65	—	—	—	—	65	—	—
24	—	—	—	—	61	36	—	—	—	—	—	—
25	—	—	—	—	61	—	—	—	—	—	—	—

VIOAR – *Viola arvensis*; CENCY – *Centaurea cyanus*; LAMPU – *Lamium purpureum*; STEME – *Stellaria media*; THLAR – *Thlaspi arvense*; VERPE – *Veronica persica*; GALAP – *Galium aparine*; ANTAR – *Anthemis arvensis*; BRSNN – *Brassica napus*; CAPBP – *Capsella bursa-pastoris*; MATIN – *Tripleurospermum inodorum*; MATCH – *Matricaria chamomilla*.

All trials were carried out in accordance with the principles of good experimental practices (GEPs). This experimental design is consistent with the provisions of the PP 1/181(5) standard (conduct and reporting of efficacy evaluation trials, including GEP) [37]. “The trial should form part of a trial series carried out in different regions with distinct environmental conditions and preferably in different years or growing seasons” and the requirements of the PP 1/93(3) standard (Weeds in cereals) [38].

MT-565 SG was applied by broadcast foliar spraying at 1.0 kg ha^−1^ (M + T, MCPA 550 g a.i. L^−1^ + tribenuron-methyl 15 g a.i. L^−1^), and 0.8 kg ha^−1^ with dedicated adjuvant SarBio 90 EC (SB) at 50 mL per 100 L of water, compared to MCPA-based reference products Chwastox Turbo 340 SL at the dose 2,5 L ha^−1^ (750 g a.i. ha^−1^ of MCPA and 100 g a.i. ha^−1^ of dicamba), Premier D 750 SL at the dose 1.25 L ha^−1^ (825 g a.i. ha^−1^ of MCPA and 112.5 g a.i. ha^−1^ of dicamba) and Chwastox 300 SL at the dose 3.0 L ha^−1^ (900 g a.i. ha^−1^ of MCPA), already registered to control weeds in winter cereals. Herbicides were sprayed using backpack plot sprayers with flat fan nozzles, calibrated to deliver water volumes ranging from 200 to 300 L ha^−1^ aqueous solution, and the plot size of trials varied between 12 and 21 m^−2^. The experiments were designed as a complete randomized block with four replications, and the untreated control was included in the experimental design. Herbicide application times were in spring. Crop stages at application are presented in Table 1.

Before application and during efficacy assessments, the weed population in untreated control plots was recorded in absolute terms by recording the density (number of plants m^−^²) of each weed. The percentage efficacy of the tested products was visually assessed in each treated plot by comparison to the untreated control plot. The results were expressed simply as a percentage according to an inverted scale to express the percentage of weed control (0% = no weed control, 100% = full weed control). The assessments to evaluate the efficacy effect of the test products on weeds were carried out 14 and 28 days after treatment (DAT).

Analyses of the plant communities were carried out before the herbicide application on permanent research plots, which were homogeneous plant patches of cereals. The total number of species in all plots was determined, and the weed species in the studied areas were marked. The species composition of weed communities and the number of plants of each species from the untreated control plots were used to assess the biodiversity. Simpson (*D*) = 1 − ∑*p*_*i*_; Shannon–Wiener (*H*′) = \[-{\sum }_{i=1}^{k}({p}_{i}\mathrm{ln}{p}_{i});]\] Margalef’s (*D*_Mg_) = (*S* − 1)/ln *N* [39,40]; and Berger–Parker (*d*) = *n*_max_/*N* [41], where *k* is the number of categories, *p*_*i*_ is the share of each species in the sample, *S* is the number of species, *N* is the total number of individuals in the sample, and *N*_max_ is the number of individuals of the most abundant species. Frequency (*F*) and relative frequency (RF) were calculated using the formulas: *F*(%) = (number of sampling units in which species occurred/total number of sampling units) × 100 and RF = (number of target species occurred/number of all species occurred) × 100. Using Sorenson’s index of similarity, a comparison of dominance among weed communities between trials was made, according to the formula [42] *S* = (2 *J*/*A* + *B*) × 100, where *S* is an index of association between treatments *A* and *B*, *J* is the number of species common in both treatments *A* and *B*, *A* is the number of species present in treatment *A*, and *B* is the number of species present in treatment *B*.

Statistica 13 software (StatSoft Poland) was used to calculate statistical analysis, and Tukey’s honest significant difference test was used to separate treatment means (*P* = 0.05). Percent rating of weed control was arc-sine transformed prior to analysis to correct for unequal variance. Data in tables are reported as non-transformed. Data were pooled only by treatment because the random effects of treatment, year, and their interactions were not significant.


**Ethical approval:** The conducted research is not related to either human or animal use.

## 3 Results

In the field experiments, 12 species of broadleaved weeds were recorded: *Anthemis arvensis* L. (ANTAR), *Brassica napus* L. (BRSNN), *Capsella bursa-pastoris* (L.) Medik (CAPBP), *Tripleurospermum maritimum* (L.) (MATIN), *Viola arvensis* Murr. (VIOAR), *Centaurea cyanus* L. (CENCY), *Lamium purpureum* L. (LAMPU), *Stellaria media* (L.) Vill. (STEME), *Thlaspi arvense* L. (THLAR), *Veronica persica* Poir. (VERPE), *Galium aparine* L. (GALAP), and *Matricaria chamomilla* L. (MATCH) (Table 2).

RF determines the results of competition, and for ANTAR, it ranged from 15.9 to 30.5%, BRSNN 17.3–35.3%, CAPBP 16.6–100%, CENCY 13.5–55.6%, GALAP 15.2–43.5%, LAMPU 15.3–26.9%, MATCH 11.6–28.6%, MATIN 12.7–20.5%, STEME 2.4–35.1%, THLAR 17.6–36.6%, VERPE 17.8–35.2%, and VIOAR 17.3–100.0% (Table 3). The values of the Margalef indicator (*D*_Mg_) indicated that weed communities varied between 0.68 and 0.38. Also, the Shannon diversity index (H′) varied widely from 0.0 to 0.778 (Table 3). The share of individual species in the community described by the Simpson index (D) ranged from 0.0 to 0.89. The dominance of the most abundant species is measured using the Berger–Parker index (*d*), which varied from 0.166 to 1.0, but values mostly ranged from 0.166 to 0.455 (20 field studies) than 0.556–1.0 (5 studies) (Table 3).

**Table 3:** Weed species occurring in experimental fields

Trial no.	Weed species – EPPP codes, RF %	*D*_Mg_	*H*′	*D*	*d*
1	ANTAR, 19.2; BRSNN, 22.4; CAPBP, 16.9; MATIN, 19.2; VIOAR, 22.4	2.67	0.697	0.82	0.224
2	ANTAR, 15.9; CAPBP, 16.6; CENCY, 16.6; LAMPU, 16.6; STEME16.6; THLAR 17.6	3.38	0.778	0.89	0.166
3	ANTAR, 17.7; BRSNN, 35.3; THLAR, 18.7; VIOAR, 28.3	2.07	0.584	0.76	0.353
4	ANTAR, 30.5; BRSNN, 22.9; CENCY, 26.2; VIOAR, 20.4	1.88	0.597	0.76	0.305
5	ANTAR, 19.3; CENCY, 21.4; STEME, 20.0; VERPE, 20.0; VIOAR, 19.3	2.37	0.699	0.82	0.214
6	BRSNN, 17.3; GALAP, 20.2; LAMPU,15.3; STEME, 29.8; VIOAR, 17.3	2.60	0.686	0.81	0.298
7	BRSNN, 17.8; CAPBP, 19.8; CENCY, 14.0; MATIN, 12.7; STEME, 17.8; VERPE, 17.8	3.14	0.773	0.85	0.198
8	BRSNN, 18.6; CENCY, 13.5; MATIN, 12.8; VERPE, 35.2; VIOAR, 19.9	2.51	0.667	0.79	0.352
9	CAPBP, 100	0.0	0.0	0.0	1.0
10	CAPBP, 43.5; CENCY, 30.4; MATCH, 26.1	1.47	0.467	0.68	0.435
11	CAPBP, 44.4; CENCY 55.6	0.80	0.298	0.52	0.556
12	CENCY, 25.3; GALAP, 15.2; MATCH, 11.6; STEME, 2.4; VIOAR, 45.5	1.67	0.579	0.69	0.455
13	CENCY, 19.3; LAMPU, 21.2; MATIN, 20.5; THLAR, 18.5; VERPE, 20.5	2.83	0.698	0.83	0.224
14	CENCY, 22.3; GALAP, 18.2; MATCH, 17.1; STEME, 17.9; VIOAR, 24.4	1.83	0.695	0.80	0.244
15	CENCY, 21.7; GALAP, 21.7; VERPE, 21.7; VIOAR, 34.8	2.20	0.592	0.77	0.348
16	CENCY, 40.0; MATCH, 26.7; VIOAR, 33.3	1.35	0.471	0.68	0.400
17	CENCY, 37.4; MATIN, 20.5; THLAR, 21.1; VERPE, 21.1	1.93	0.586	0.75	0.374
18	CENCY, 33.3; MATCH, 28.6; VIOAR, 38.1	1.51	0.474	0.70	0.381
19	GALAP, 28.4; STEME, 35.1; THLAR, 36.6	1.32	0.475	0.68	0.366
20	GALAP, 43.5; LAMPU, 20.9; THLAR, 35.6	1.43	0.459	0.67	0.435
21	LAMPU, 26.9; STEME, 28.5; VERPE, 16.8; VIOAR, 27.7	1.68	0.593	0.75	0.285
22	VIOAR, 100	0.0	0.0	0.0	1.0
23	VIOAR, 77.8; VERPE, 22.2	0.70	0.230	0.36	0.444
24	VIOAR, 62.1; CENCY, 37.9	0.68	0.288	0.49	0.629
25	VIOAR, 100	0.0	0.0	0.0	1.0

RF – relative frequency; *D*_Mg_ – Margalef diversity index; *H*′ – Shannon index; *D* – Simpson’s index of diversity; *d* – Berger-Parker dominance index.

VIOAR – *Viola arvensis*; CENCY – *Centaurea cyanus*; LAMPU – *Lamium purpureum*; STEME – *Stellaria media*; THLAR – *Thlaspi arvense*; VERPE – Veronica persica; GALAP – *Galium aparine*; ANTAR – *Anthemis arvensis*; BRSNN – *Brassica napus*; CAPBP – *Capsella bursa-pastoris*; MATIN – *Tripleurospermum inodorum*; MATCH – *Matricaria chamomilla*.

**Figure 2:**
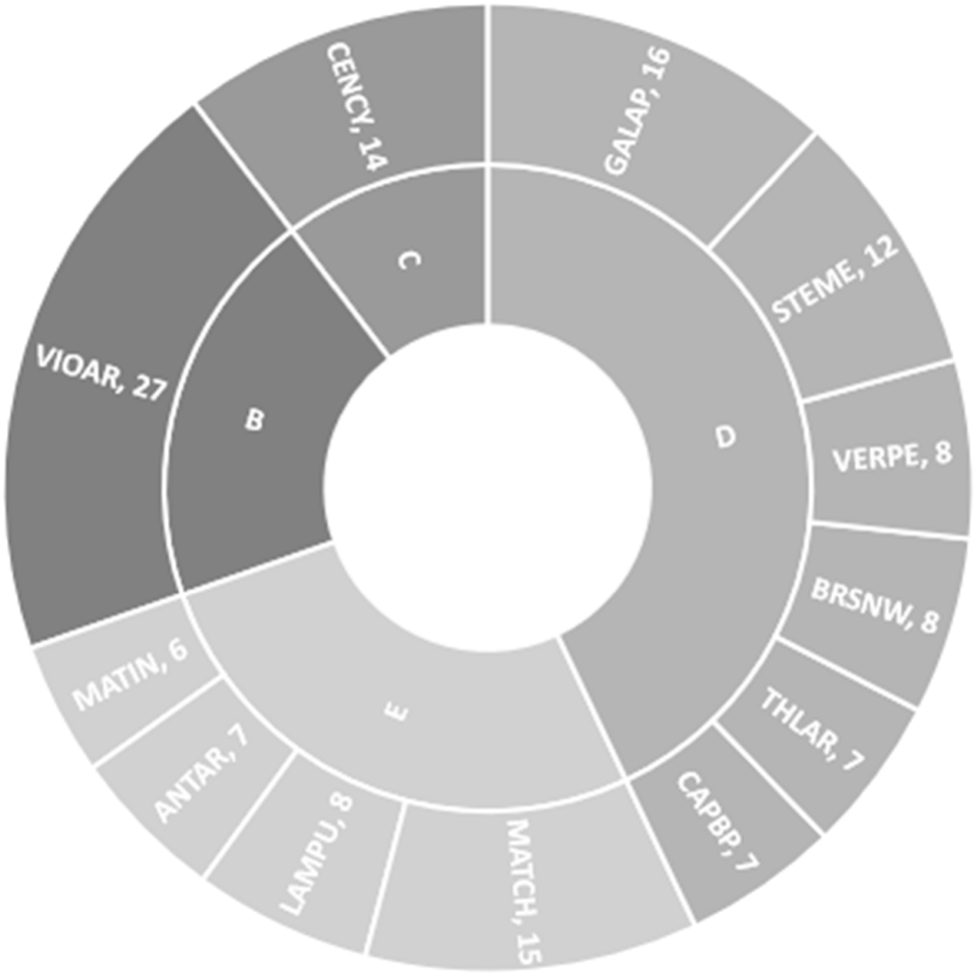
Frequency class distribution of weed species (frequency classes: *A* = ≥80, *B* = 61–80.9; *C* = 41–60.9; *D* = 21–40.9; *E* = ≤20%), and average weed density (no. m2) in the years 2016–2017. VIOAR – *Viola arvensis*; CENCY – *Centaurea cyanus*; LAMPU – *Lamium purpureum*; STEME – *Stellaria media*; THLAR – *Thlaspi arvense*; VERPE – *Veronica persica*; GALAP – *Galium aparine*; ANTAR – *Anthemis arvensis*; BRSNN – *Brassica napus*; CAPBP – *Capsella bursa-pastoris*; MATIN – *Tripleurospermum inodorum*; and MATCH – *Matricaria chamomilla.*

Weed species occurring during field studies were grouped into four frequency classes: *B* = 61–80.9; *C* = 41–60.9; *D* = 21–40.9; and *E* = ≤20% (Figure 2). Only one species, VIOAR, was recorded in frequency class B (the most frequent species recorded in the study), one in *C*, six in *D*, and four in *E* class. In our study, higher values were foremost obtained in higher frequency classes.

The Sorenson’s index similarity (*S*) varied from 66.3 to 100% and indicates the close similarity in weed species between 1 with 3, 4, 22, and 25; 3 with 1, 4, 22 and 25; 4 with 1, 3, 5, 12, 22, and 24; 5 with 4, 12, 15, 21, and 22; 6 with 21 and 22; 7 with 8; 8 with 7, 15, 17, 22, and 23; 10 with 11; 11 with 10; 12 with 4, 5, 14, 15, 16, 18, and 23; 14 with 12, 15, 16, and 18; 15 with 5, 8, 12, and 14; 16 with 12, 14, 18, 22, 24, and 25; 17 with 8; 18 with 12, 14, 16, 22, 24, and 25; 19 with 20; 21 with 5, 6, and 22; 22 with 1, 3, 4, 5, 6, 8, 16, 18, 21, 23, and 25; 23 with 8, 22, 24, and 25; and 25 with 1, 3, 16, 18, 22, 23, and 24 (Table 4).

**Table 4:** Sorenson’s index of similarity (*S*) in weed species among trial sites

Trial no.	1	2	3	4	5	6	7	8	9	10	11	12	13	14	15	16	17	18	19	20	21	22	23	24	25
1	—	34.4	72.1	69.4	39.8	37.9	54.0	54.3	31.0	28.2	27.0	42.9	19.8	24.1	25.6	27.7	19.9	28.7	0.0	0.0	25.9	83.7	48.0	41.5	66.8
2	34.4	—	34.9	26.7	56.3	39.6	50.9	14.9	31.2	50.8	58.2	28.3	54.6	39.1	18.8	28.3	47.3	23.5	53.7	44.4	48.1	0.0	0.0	27.1	0.0
3	72.1	34.9	—	76.9	41.3	47.7	25.1	49.0	0.0	0.0	0.0	43.7	18.6	25.0	31.2	30.9	20.1	32.5	28.3	26.7	27.9	81.6	52.4	45.4	71.1
4	69.4	26.7	76.9	—	67.6	39.2	40.5	60.8	0.0	27.8	35.4	67.5	23.5	46.7	50.2	58.2	31.5	55.2	0.0	0.0	24.9	85.2	43.7	69.3	61.5
5	39.8	56.3	41.3	67.6	—	18.5	39.7	64.2	0.0	24.3	30.6	71.2	40.8	63.7	66.3	53.1	48.5	49.9	26.0	0.0	66.9	76.3	60.8	62.7	56.5
6	37.9	39.6	47.7	39.2	18.5	—	41.0	36.7	0.0	0.0	0.0	63.7	17.9	61.8	45.1	24.8	0.0	25.2	56.5	17.7	75.7	81.3	43.8	37.7	61.6
7	54.0	50.9	25.1	40.5	39.7	41.0	—	71.2	31.1	48.6	54.6	28.3	50.8	38.5	36.1	25.3	60.9	20.7	25.7	0.0	41.6	0.0	9.0	24.2	0.0
8	54.3	14.9	49.0	60.8	64.2	36.7	71.2	—	0.0	19.8	26.7	65.6	61.0	44.0	72.2	50.7	69.8	46.7	0.0	0.0	48.7	80.0	71.9	61.7	61.3
9	31.0	31.2	0.0	0.0	0.0	0.0	31.1	0.0	—	55.8	59.0	0.0	0.0	0.0	0.0	0.0	0.0	0.0	0.0	0.0	0.0	0.0	0.0	0.0	0.0
10	28.2	50.8	0.0	27.8	24.3	0.0	48.6	19.8	55.8	—	85.4	38.6	24.5	41.7	26.1	62.3	34.6	59.1	0.0	0.0	0.0	0.0	0.0	0.0	0.0
11	27.0	58.2	0.0	35.4	30.6	0.0	54.6	26.7	59.0	85.4	—	27.4	34.2	25.8	36.6	45.8	43.5	43.6	0.0	0.0	0.0	0.0	0.0	44.7	0.0
12	42.9	28.3	43.7	67.5	71.2	63.7	28.3	65.6	0.0	38.6	27.4	—	24.7	100	85.3	84.3	26.8	83.4	23.0	17.8	49.6	63.2	48.7	73.9	53.5
13	19.8	54.6	18.6	23.5	40.8	17.9	50.8	61.0	0.0	24.5	34.2	24.7	—	9.9	7.6	6.2	19.9	4.5	6.0	9.1	12.3	0.0	6.2	5.8	0.0
14	24.1	39.1	25.0	46.7	63.7	61.8	38.5	44.0	0.0	41.7	25.8	100	9.9	—	66.7	69.8	25.2	68.2	40.9	21.8	46.3	57.2	32.4	55.2	40.6
15	25.6	18.8	31.2	50.2	66.3	45.1	36.1	72.2	0.0	26.1	36.6	85.3	7.6	66.7	—	19.0	16.2	16.0	7.7	8.9	18.7	46.3	22.1	23.0	25.5
16	27.7	28.3	30.9	58.2	53.1	24.8	25.3	50.7	0.0	62.3	45.8	84.3	6.2	69.8	19.0	—	38.6	100	0.0	0.0	29.6	86.5	54.4	86.4	72.2
17	19.9	47.3	20.1	31.5	48.5	0.0	60.9	69.8	0.0	34.6	43.5	26.8	19.9	25.2	16.2	38.6	—	35.9	28.5	27.1	18.4	0.0	21.6	37.6	0.0
18	28.7	23.5	32.5	55.2	49.9	25.2	20.7	46.7	0.0	59.1	43.6	83.4	4.5	68.2	16.0	100	35.9	—	0.0	0.0	30.4	90.6	60.4	88.0	79.4
19	0.0	53.7	28.3	0.0	26.0	56.5	25.7	0.0	0.0	0.0	0.0	23.0	6.0	40.9	7.7	0.0	28.5	0.0	—	71.1	30.8	0.0	0.0	0.0	0.0
20	0.0	44.4	26.7	0.0	0.0	17.7	0.0	0.0	0.0	0.0	0.0	17.8	9.1	21.8	8.9	0.0	27.1	0.0	71.1	—	25.2	0.0	0.0	0.0	0.0
21	25.9	48.1	27.9	24.9	66.9	75.7	41.6	48.7	0.0	0.0	0.0	49.6	12.3	46.3	18.7	29.6	18.4	30.4	30.8	25.2	—	75.3	61.5	38.8	57.1
22	83.7	0.0	81.6	85.2	76.3	81.3	0.0	80.0	0.0	0.0	0.0	63.2	0.0	57.2	46.3	86.5	0.0	90.6	0.0	0.0	75.3	—	95.9	24.5	100
23	48.0	0.0	52.4	43.7	60.8	43.8	9.0	71.9	0.0	0.0	0.0	48.7	6.2	32.4	22.1	54.4	21.6	60.4	0.0	0.0	61.5	95.9	—	69.6	91.3
24	41.5	27.1	45.4	69.3	62.7	37.7	24.2	61.7	0.0	0.0	44.7	73.9	5.8	55.2	23.0	86.4	37.6	88.0	0.0	0.0	38.8	24.5	69.6	—	87.0
25	66.8	0.0	71.1	61.5	56.5	61.6	0.0	61.3	0.0	0.0	0.0	53.5	0.0	40.6	25.5	72.2	0.0	79.4	0.0	0.0	57.1	100	91.3	87.0	—

MT-565 SG at both rates controlled VIOAR in the range of 35.2–39.6% at the first assessment 14 DAT and in the range of 58.4–67.3% at the final assessment 28 DAT. A slightly worse control of VIOAR was observed in both assessments after the use of reference products based on MCPA, and at the final assessment, 28 DAT moderate control was observed at 62.3%. A similar relationship can be noticed in the control of *Matricaria chamomilla* (MATCH) and CENCY. Slightly better control of MATCH and CENCY after 14 DAT was observed after using MT-565 SG at a dose of 1.0 kg ha^−1^ than after reference products and good control of both weeds were continued, giving at the final assessment 28 DAT results between 85.6 and 89.9% in the case of MT-565 SG (1.0 kg ha^−1^) and 82.0–86.4% in the case of reference products. Stellaria media was moderately controlled at the first assessment by all products (48.4–55.3%), giving at the final assessment very good control by MT-565 SG at a dose of 1.0 kg ha^−1^ (92.7%) and reference products based on MCPA (92.6%). GALAP was well controlled by a higher dose of MT-565 SG (67 vs 77.9%) at the final assessment 28 DAT. A comparable good control of GALAP (28 DAT) was recorded for MCPA-based products at 81.4%. Less numerously occurring broadleaved weed species were moderately controlled (ANTAR, BRSNN, CAPBP, LAMPU, MATIN, THLAR) by MT-565 SG at a dose of 1.0 kg ha^−1^ at the first assessment 14 DAT by 44.5–57.7% and slightly better after use of reference products (36.6–51.8%). Only control of VERPE was more difficult by MT-565 SG at 14 DAT and was less effective in comparison to the reference product (37.8 vs 41.1%). A lower dose of MT-565 SG controlled less dense weeds in the range of 32.5–52.3% at 14 DAT. Also, during the final assessment at 28 DAT, a similar relationship can be observed; a lower dose of MT-565 SG eliminates less effectively weeds up to 10.0% in the case of *Thlaspi arvense* (THLAR) and comparison to a higher dose of MT-565 SG and reference products. The higher dose of MT-565 SG provided an overall good control (79.3–94.1%) against less dense weeds, and biological efficacy was better than the reference products in the case of control VERPE (79.3 vs 77.1%), THLAR (90.8 vs 86.8%), LAMPU (94.3 vs 90.6%), and CAPBP (84.6 vs 79.8%) (Table 5).

**Table 5:** Influence of herbicides MT-565 SG and reference products on broadleaved weed control in winter cereals – Poland 2016–2017

Percent of weed control (number of sites)														
No.	Herbicide	Dose per ha	ANTAR (5)	BRSNN (6)	CAPBP (6)	CENCY (15)	GALAP (6)	LAMPU (5)	MATCH (5)	MATIN (8)	STEME (8)	THLAR (6)	VERPE (8)	VIOAR (16)
Control (average no. per sqm.)		–	7.5	7.7	7.1	13.6	16.3	7.5	15.0	5.7	11.8	7.3	8.1	27.3
**First assessment 14 DAT**														
1	MT-565 SG + ad.	0.8 kg + 50 mL per 100 L	45.5	45.2	52.3	46.3	40.4	43.8	73.1	40.5	49.8	40.6	32.5	35.2
2	MT-565 SG + ad.	1.0 kg + 50 mL per 100 L	49.8	47.5	57.7	52.3	44.8	46.0	76.6	44.5	55.3	52.5	37.8	39.6
3	Ref. products	2.5 L/1.25 L/3.0 L	51.8	49.2	47.1	49.5	45.8	42.8	70.0	36.6	48.4	49.2	41.1	34.1
HSD 0.05			3.4	ns	4.7	ns	5.1	ns	4.0	7.4	6.5	4.7	2.8	3.9
**2nd assessment 28 DAT**														
1	MT-565 SG + ad.	0.8 kg + 50 mL per 100 L	82.5	86.7	76.7	79.8	67.0	87.8	84.3	80.8	89.4	80.8	72.4	58.4
2	MT-565 SG + ad.	1.0 kg + 50 mL per 100 L	88.9	92.2	84.6	85.6	77.9	94.3	89.9	87.5	92.7	90.8	79.3	67.3
3	Ref. products	2.5 L/1.25 L/3.0 L	91.7	93.0	79.8	82.0	81.4	90.6	86.4	88.8	92.6	86.8	77.1	62.3
HSD 0.05			3.0	2.2	5.2	4.1	4.3	2.8	4.1	2.9	2.8	1.8	2.8	5.1

DAT – days after treatments; ad. – adjuvant; ns – nonsignificant; ANTAR – *Anthemis arvensis*; BRSNN – *Brassica napus*; CAPBP – *Capsella bursa-pastoris*; CENCY – *Centaurea cyanus*; GALAP – *Galium aparine*; LAMPU – *Lamium purpureum*; MATCH – *Matricaria chamomilla*; MATIN – *Tripleurospermum inodorum*; STEME – *Stellaria media*; THLAR – *Thlaspi arvense*; VERPE – *Veronica persica*; VIOAR – *Viola arvensis*.

## 4 Discussion

Postemergence herbicide efficacy is determined by many factors, not only by active ingredients or formulation. Factors like timing of application, rainfast period, temperature, relative humidity, and water quality (turbidity, hard water, pH) are critical for the best results from post-emergent herbicides [43]. The weed species pool is usually higher in the organic than in the conventional treatment [44], and their composition is still one of the most important factors affecting the final result of weed control [45]. In our study, the Margalef indicator (*D*_Mg_) values measuring the evenness indicated that weed communities varied from poor to great biodiversity. Also, the Shannon diversity index (*H*′) varied widely, indicating great diversity of weed communities between regions in Poland. The share of individual species in the community described by the Simpson index (*D*) expresses the probability of meeting two individuals belonging to the same species, ranging widely from communities consisting of only one species to values close to the greatest diversity. It should be concluded that the results obtained indicate mostly a rather greater diversity of the weed community in cereals. The dominance of the most abundant species measured by the Berger–Parker index (*d*) varies from 0 (the highest diversity) to 1 (monoculture), indicating the high diversity of weed communities in cereals in Poland. Weed species occurring during field studies were grouped into frequency classes, which give an approximate indication of the homogeneity and heterogeneity of species [46], and high values in lower frequency and low values in higher frequency classes state a high degree of florists heterogeneity [47]. In our study, higher values were foremost obtained in higher frequency classes, which indicates that rather a medium degree of florist heterogeneity existed in study fields.

Weed community composition usually varies between sites, and Sorenson’s index similarity indicates the species similarity in weed communities between trial sites. Our results mostly confirm high similarity in weed species between sites, but in some cases, lower similarity, indicating there was some variability in weed species community or moderate similarity; in others (index below 40%), showing that there was some variability in weed species, even complete when index was 0.

Weeds can contribute to a significant reduction in the yield of winter cereals [48]. At the same time, they can hinder their harvest [49]. It is therefore important to control them properly. Our results indicate that the use of both the MCPA + tribenuron-methyl mixture and reference products may significantly limit their development. Other authors also indicate that the substances used contribute to the appropriate control of weeds in the cultivation of winter cereals [50,51,52]. The test herbicide contains a mixture of MCPA + tribenuron-methyl. These substances exhibit different mechanisms of action. The use of such mixtures is important for the prevention of weed resistance [27]. In addition, the use of herbicide mixtures allows the control of a wide spectrum of weed species [53]. The tested mixture also showed high effectiveness in the case of autumn application [54]. Khalil et al. [55] showed that the use of herbicide mixtures in wheat cultivation gives better results than the application of single plant protection products. Bobrovsky et al. [56] showed that the use of herbicide mixtures allows for a significant reduction of the negative impact of weeds on the level of wheat yield. However, tank mixtures of different herbicides may contribute to antagonisms that lead to impaired herbicide uptake and translocation and [57], consequently, their effectiveness. Therefore, a good solution is to use tested factory mixtures.

## 5 Conclusions

Chemical weed control is the most common and effective weed control method. The first element in achieving the appropriate efficacy of the herbicide treatment is matching the appropriate plant protection product to the weeds present. The use of mixtures of active substances allows for the control of a wide spectrum of weed species, which was confirmed in the described experiment. In addition, it is an important issue in the context of preventing weed resistance to herbicides. The test herbicide contains a mixture of MCPA + tribenuron-methyl, which showed high effectiveness in all locations. It has contributed to significant control of many weed species. The obtained results indicate that this herbicide can be successfully used during spring application in cereal cultivation.
